# The Use of Artificial Intelligence in the Management of Neurodegenerative Disorders; Focus on Alzheimer’s Disease

**DOI:** 10.31661/gmj.v12i.3061

**Published:** 2023-09-03

**Authors:** Khazar Ghasempour Dabbaghi, Zahra Khosravirad, Sheida Jamalnia, Rahil GhorbaniNia, Fatemeh Mahmoudikohani, Habib Zakeri, Solmaz Khastehband

**Affiliations:** ^1^ School of Medicine, Tabriz University of Medical Sciences, Tabriz, Iran; ^2^ Department of Quality Improvement in Kowsar Hospital, Shiraz, Iran; ^3^ Department of Nursing and Midwifery, Kazeroun Branch, Islamic Azad University, Kazeroun, Iran; ^4^ Noncommunicable Disease Research Center, Bam University of Medical Science, Bam, Iran; ^5^ Department of Midwifery, School of Nursing and Midwifery, Bam University of Medical Science, Bam, Iran; ^6^ Research Center for Neuromodulation and Pain, NAB Pain Clinic, Shiraz University of Medical Sciences, Shiraz, Iran; ^7^ Department of Educational Management, Islamic Azad University-south Tehran Branch, Tehran, Iran

**Keywords:** Artificial Intelligence, Neurodegenerative Diseases, Alzheimer

## Abstract

Recent advances in artificial intelligence (AI) have shown great promise in the diagnosis, prediction, treatment plans, and monitoring of neurodegenerative disorders. AI algorithms can analyze huge quantities of data from numerous sources, including medical images, quantifiable proteins in urine, blood, and cerebrospinal fluid (CSF), genetic information, clinical records, electroencephalography (EEG) signals, driving behaviors, and so forth. Alzheimer’s disease (AD) is one of the most common neurodegenerative disorders that progressively damage cognitive abilities and memory. This study specifically explores the possible application of AI in the diagnosis, prediction, monitoring, biomarker or drug discovery, and classification of AD.

## Introduction

Artificial intelligence (AI) has revolutionized numerous industries, and the field of medical science is no exception. In this age of information and technology, the integration of AI into medical sciences has brought about a new era of healthcare, offering significant benefits to patients, healthcare professionals, and the industry as a whole [1][2][3]. AI can process large amounts of medical data and identify patterns that would otherwise be difficult for humans to detect. With the ability to learn from vast amounts of medical data, AI has the potential to transform medical research and personalized medicine. AI algorithms and techniques can help healthcare professionals analyze large datasets and provide insights that were previously impossible to obtain [4][5]. Neurodegenerative diseases are a range of conditions affecting the neurons in the brain and spinal cord and cause their degeneration. This can lead to a variety of symptoms, including cognitive impairment, movement disorders, and psychiatric symptoms [6][7]. Neurodegenerative diseases affect millions of people quality of life. The exact causes of neurodegenerative diseases are not fully understood, but they are supposed to be developed by a combination of lifestyle, environmental, and genetic factors. There are many types of neurodegenerative diseases, each with its unique set of symptoms and characteristics. The most common neurodegenerative diseases are Alzheimer’s disease (AD), Parkinson’s disease, Huntington’s disease, and amyotrophic lateral sclerosis (ALS) [8][9].

AD is a chronic, incurable brain disease that gradually damages cognition and memory. The majority of dementia cases are caused by AD, which typically affects older people and age is one of its risk factors. The three stages of AD development are as follows [10][11]. The first stage is the pre-clinical AD stage which patients are clinically asymptomatic, although cerebral amyloidosis is present in the brain as a quantifiable change. The second stage is mild cognitive impairment (MCI) which develops at the following stage and the affected individuals’ families may be the only ones to discover minor symptoms and signs of brain abnormalities. Patients acquire dementia brought on by AD in the third stage, with symptoms severe enough to limit everyday functioning [10][12].

Recent advances in AI have shown great promise in the diagnosis, prediction, treatment plans, and monitoring of the progression of neurodegenerative disorders [13]. AI algorithms can analyze large amounts of data from various sources, including medical images, genetic information, and clinical records, to identify early disease markers, develop personalized treatment plans, and predict disease progression of neurodegenerative disorders [14][15].

This review presents potential applications of AI and its subsets in the diagnosis, prognosis, treatment, follow-up, biomarker discovery, drug discovery, and prediction of AD as one of the most common neurodegenerative disorders.

1. Diagnosis

AI has been applied to many data sources to diagnose AD including medical images, quantifiable proteins in urine, blood, and cerebrospinal fluid (CSF), genetic information, clinical records, electroencephalography (EEG) signals, driving behaviors, and so forth [16].

Medical imaging is one of the most crucial methods in the assessment of patients with brain pathology in diseases including AD. Three basic categories of imaging techniques are employed: molecular imaging, including positron emission tomography (PET), single photon emission computed tomography (SPECT), and functional MRI (fMRI); functional imaging, including fMRI scans and PET; and structural imaging, including computed tomography (CT) scans and magnetic resonance imaging (MRI).

AI has been used by researchers to automatically detect complicated patterns in images and make quantitative assessments of radiology data. AI can aid in enhancing both the accuracy and effectiveness of image analysis. Mistakes and delays in diagnosis due to radiologist errors can lead to poor patient outcomes.

The use of AI in radiology is expected to accomplish not only faster and more cost-effective image interpretation but also more reliable image interpretation in the current era of digital imaging databases and electronic health record systems [17][18].

One way to use AI to diagnose AD is to analyze quantifiable proteins in biological samples like serum and CSF. One example of an AI-based approach to analyze CSF is the use of machine learning algorithms to identify patterns of protein expression that are indicative of AD.

Researchers have identified several key proteins that are present in abnormal levels in the CSF of Alzheimer’s patients, including beta-amyloid, tau, and neurofilament light chain [19][20]. The study of Eleonora Ficiarà et al. [21] investigated the possible impact of iron on early diagnosis and therapy possibilities, both in serum and CSF. Non-demented neurological controls, frontotemporal dementia, mild cognitive impairment, and AD patients were included in the study. Utilizing iron-related data, the use of machine learning techniques such as multiclassification algorithms and clustering analysis revealed a new possible stratification of patients. The findings confirmed that iron dysregulation had a significant impact on the pathogenesis of dementia maybe through interactions with biomarkers like tau protein and amyloid-beta [21].

Language and speech skills are valuable sources of clinical data in AD, as it drops simultaneously with neurodegeneration. Recently, collecting language and voice data in various ways and employing computational speech processing for diagnosis, prediction, or progression using AI, is introduced as a new approach in AD research [22]. Alexandra König et al. [23] designed a study to assess the feasibility of employing automatic speech analysis to diagnose MCI and early-stage AD. Several short cognitive vocal tasks were performed by patients with MCI or AD and also healthy elderly control volunteers.

Using speech signal processing techniques, the recorded voices were analyzed and the initial vocal indicators were extracted. Then, the vocal markers were assessed for their "power" to distinguish between AD, MCI, and control groups. The next phase involved utilizing machine learning approaches to create automatic classifiers for detecting MCI and AD and then verifying the detection accuracy. Automatic audio analyses had the following classification accuracy: 80% ± 5% between AD and MCI, 87% ± 3% between AD and controls; and 79% ± 5% between MCIs and controls, proving its assessment utility. The main limitations of the field are a degree of disconnect between study aims and clinical applications, limited comparability of results, and poor standardization [23][24].

2. Early Stage Diagnosis

Clinically, early or asymptomatic AD diagnosis has a significant impact since it enables early intervention [25]. Several studies used multi-modality imaging including MRI, fluorodeoxyglucose-positron emission tomography (FDG-PET), tau-PET, amyloid-PET, and AI for diagnosis and prognosis of early stages (at the MCI or preclinical stages) of AD. These imaging techniques can be broadly divided into two categories: those used to detect neuronal damage and those used to detect amyloid positive. Several studies used FDG-PET to examine the connection between glucose metabolism and amyloid deposition in people with normal cognitive function. In people with severe amyloid deposition and normal cognitive function, several studies discovered hypometabolism [26].

The preclinical stage of AD was predicted by Haifeng Chen et al. [27] using a machine-learning approach based on the multimodal connectome. The participants were 110 healthy controls and preclinical AD individuals.

Morphological, anatomical, and functional networks were created using multimodal imaging data. To predict preclinical patients, these networks were combined with a multiple kernel learning-support vector machine. Results indicated that the characteristics identified from the multimodal network achieved an accuracy of 88.73% based on the integration of the three modalities.

3. Predicting MCI Conversion to AD and Time to Conversion

Several researchers attempted to predict the possibility and duration required for patients with MCI to convert to AD [28]. According to a study by Tingting Zhang et al. [29], brain connection network features and cortical thickness parameters obtained from structural MRI and resting state fMRI (rs-fMRI) could distinguish between patients with MCI converter and those with mild cognitive impairment non-converters (MCInc/AD). The converted sensitivity brain regions of the two patient groups (MCInc vs. MCIc) and the same group of patients (MCIc vs. AD) may differ and can accurately predict the conversion of MCI to AD, according to the findings.

Predicting how long it will take people with MCI to develop AD is a complex task, as the rate of disease progression varies greatly from person to person. However, AI can be used to develop predictive models based on various factors that have been shown to influence the risk of conversion from MCI to AD [30].

Some researchers make an effort to forecast how long it will take for people with MCI to transition to AD. Simon F. Eskildsen et al. [31] introduced a novel approach to subgroup MCI subjects in terms of "time to conversion". They showed that they could predict conversion from baseline within 3 years with an accuracy of 74% when utilizing the stratified classifiers in a clinically applicable manner. Although this prediction had a low sensitivity (64%), it had a high specificity (84%). To be therapeutically useful, the long-term prediction’s sensitivity must be increased to maximize the benefits of potential neuroprotective treatments.

4. AD Staging and Classification

Despite many types of research evaluating cognitive monitoring, most address only the presence or absence of cognitive impairments.

AI can use medical images to detect disease stages. For example, Robin Ghosh et al. [32] examined datasets containing around 6,400 MRI images, each segregated into the severity of AD stages: moderate dementia, non-dementia, very mild dementia, and mild dementia. The convolutional neural network (CNN) technique was utilized to classify the dementia stages in each patient using these four image criteria.

Using a CNN-based in silico model, the authors accurately predicted and classified the various AD phases with 97.19% accuracy.

Another popular research area is recognizing the distinction between normal control (NC), MCI, and AD. Edward Challis et al. [33] used the Gaussian process classification of AD and MCI from resting-state fMRI. They tested the effectiveness of a particular multivariate statistical machine-learning technique for patient classification using patterns of resting-state functional connectivity in the brain. The applied model distinguished between amnesic mild cognitive impairment (CI) and healthy individuals with 75% accuracy and between AD and amnesic MCI subjects with 97% accuracy.

Qing Li et al. [34] tried multi-feature kernel discriminant dictionary learning to classify individuals without cognitive impairment (CI), MCI, and AD. They attained classification accuracy of 98.18% when comparing CU to AD, 78.50% when comparing CU to MCI, and 74.47% when comparing MCI to AD. Overall, AI holds great promise for improving our ability to stage AD and to develop personalized treatment plans for patients at different stages of the disease. However, these approaches are still in the early steps of development and require further validation in larger, longitudinal studies.

5. AD Biomarker Discovery

5.1. Blood-based Biomarkers

Researchers have identified several blood-based biomarkers that are associated with AD, including levels of certain proteins and lipids [35].

Machine learning algorithms can be used to analyze large amounts of blood data to identify these biomarkers and predict the risk of developing AD. Yilmaz Ali et al. [36] carried out a study using machine learning and AI, to determine whether a group of plasma metabolites can be utilized as a practicable tool for the diagnosis of AD and MCI. They used a method combining different statistical approaches of machine learning, liquid chromatography coupled with mass spectrometry (LC-MS), and proton nuclear magnetic resonance spectroscopy (1H NMR) to find a biomarker panel that could distinguish between patients with AD and MCI and healthy controls. Five of the 212 analyzed metabolites were found to be the most useful in differentiating between AD or MCI from healthy controls. Lipid metabolism was found to be the most disrupted metabolic process in MCI and AD by univariate and pathway analyses.

5.2. Urine-based Biomarkers

AI can be used to analyze urine-based biomarkers for AD.

The effectiveness of urine metabolites as potential markers of AD and MCI was investigated using a quantitative metabolomics technique that combined mass spectrometry and 1H NMR. To create biomarker panels that were assessed using logistic regression models and support vector machine (SVM) to diagnose AD phases, least absolute shrinkage and selection operator (LASSO) and correlation-based feature selection (CFS) approaches were utilized. Eleven urine metabolites that were markedly changed in the urine of AD patients were malonate, N oxide, trimethylamine, tryptophan, 2-ketoisovalerate, 2- and 3-hydroxyisovalerate, cytosine, hippuric acid, urocanate, guanidinoacetate, and glucose. With an accuracy of 81%, a specificity of 75%, and a sensitivity value of 76%, these markers were also capable of detecting MCI [37].

5.3. MRI-based Biomarkers

Machine learning algorithms can be used to analyze MRI data to identify subtle changes in brain structure that are indicative of AD. Recently, machine learning techniques have been used to identify biomarkers associated with magnetic resonance (MR) for the in vivo differential diagnosis of AD. Using an improved machine learning algorithm, Christian Salvatore et al. [38] analyzed 162 healthy controls, 134 MCInc, 76 MCIc, and 137 AD patients from the Alzheimer’s disease neuroimaging project (ADNI) cohort. Voxels of the cerebellum, precuneus, cerebellum, gyrus rectus, basal ganglia, entorhinal cortex, and hippocampus are all crucial areas known to be heavily engaged in the pathophysiological mechanisms of AD, influenced the classification between these AD-related pre-clinical phases. Based on the identified biomarkers, the classification accuracy was 66% for MCIc vs. MCInc, 72% for MCIc vs. CN, and 76% for AD vs. CN. This type of AI-based MRI biomarker detection has the potential to greatly improve early diagnosis and treatment of AD, as it can detect subtle changes in the brain that are invisible to the human eye.

6. AD Drug Discovery

AI has the potential to significantly accelerate the drug discovery process for AD. By analyzing vast amounts of data and simulating the effects of potential drugs, AI can help identify new drug targets and accelerate the development of effective treatments for this devastating disease [39]. Three steps of the early drug discovery process (target identification, lead generation and optimization, and preclinical development) have all seen the use of AI. In target discovery, AI-based techniques have been applied to combine diverse data sets and find patterns that help comprehend the molecular processes that underlie diseases and therapeutic actions. AI algorithms help the automation and optimization of novel drug design processes and enhance scoring functions and quantitative structure-activity relationship (QSAR) models in virtual screening pipelines for lead generation and optimization. By effectively analyzing a huge quantity of chemical data, AI techniques are used in preclinical research to produce predictive models of physicochemical qualities and further optimize excretion metabolism distribution, and absorption profiles [40].

7. Limitations

Despite the many promising applications of AI in medical sciences, there are also challenges, including the need for large amounts of high-quality data and concerns around patient privacy and data security. Theoretically, adding more features should result in predictions that are more accurate, however repeatedly combining multiple data types for multi-modal representation can add irrelevant information and have a negative impact on the model's performance if done incorrectly. Some data modalities introduce noise to the subject representation since they are not appropriate for representing AD patients. Additionally, there is no assurance that adding more data modalities would lead to 100% prediction accuracy, fully representative modeling, or a comprehensive understanding of the disease [16][41].

## Conclusion

In conclusion, the integration of AI into medical sciences has brought about a new era in healthcare, and the field of neurodegenerative disorders has particularly benefitted from these advances. With the ability to analyze vast amounts of medical data from various sources, including medical images, genetic information, clinical records, EEG signals, quantifiable proteins in CSF, blood and urine, driving behaviors, etc., AI has the potential to revolutionize diagnosis, prediction, and personalized treatment of AD. AI algorithms can help healthcare professionals to reach an early diagnosis, predict and monitor disease progression, classify the disease, find new biomarkers and drugs, and personalize treatment plans. Nonetheless, the potential benefits of AI in medical sciences are significant, and AI will likely play an increasingly important role in healthcare in the coming years.

## Conflict of Interest

None.
